# Mental health status and substance abuse among medical students in Karaikal, Puducherry, India

**DOI:** 10.6026/973206300200292

**Published:** 2024-03-31

**Authors:** Ramachandran Niranjjan, S Nancy, S Gayathri, Subramaniam Arulvijayavani

**Affiliations:** 1 Department of Community Medicine, Vinayaka Mission's Medical College and Hospital, Vinayaka Mission's Research Foundation - Deemed to be University (VMRF-DU), Karaikal, Puducherry, India; 2 Department of Biochemistry, Jawaharlal Institute of Postgraduate Medical Education and Research, Karaikal, Puducherry, India

**Keywords:** Mental health, substance abuse, medical students, psychological distress, sleep deprivation

## Abstract

Mental health disorders and substance abuse are prevalent issues that significantly impact individuals and societies. Medical students are particularly vulnerable due to the intense pressures and challenges inherent in medical education. This current investigation aims to explore the mental health status and patterns of substance abuse among medical students, identifying associated factors and potential interventions. A cross-sectional study was executed with 421 undergraduate and post graduate medical students from a tertiary care centre. Multivariate logistic regression was used to determine the factors associated with psychological distress and substance abuse. Substance abuse was reported by 21.4% of participants, while 20.7% experienced psychological distress. There was a statistically significant association between substance abuse and psychological distress (p=0.005). Factors associated with psychological distress included sleep deprivation (Adjusted OR: 24.8, p=0.001), whereas factors associated with substance abuse included male gender (Adjusted OR: 2.3, p=0.001), older age, staying with friends (Adjusted OR: 1.8, p=0.04) and sleep deprivation abuse (OR: 2.0, p=0.01). This study highlights a significant occurrence of psychological distress and substance abuse among medical students. Interventions to improve mental health and reduce substance abuse among medical students should consider these associated factors, emphasizing the importance of sleep hygiene, stress management and supportive environments.

## Background:

Mental health and substance abuse issues are significant challenges faced by societies worldwide [1]. These interconnected problems have far-reaching consequences for individuals, families, and communities, necessitating urgent attention and effective strategies to address them [2]. Mental health disorders and substance abuse frequently co-occur, forming a complex nexus of challenges for individuals who are affected [1, 2]. Studies indicate that individuals identified with mental health disorders, such as depression, anxiety, and bipolar disorder, are more likely to develop substance abuse or addiction problems, and vice versa [1]. While studying addiction to substances and its negative consequences, researchers primarily focus on social groups with a higher proclivity for substance use and abuse, such as adolescents and male adults [3]. This is acceptable because they may face a variety of demanding life and social challenges, expectations, interpersonal alienation, and biological impulses, all of which contribute to their entry into drug experimentation as a form of self-medication [2]. In particular, the mental health status of medical students has garnered increasing attention in recent years due to the unique challenges they face during their education and training [4]. Medical education is known for its rigorous demands, high levels of stress, and intense pressure, which can significantly impact the mental well-being of students [3]. Medical students experience a multitude of unique stressors throughout their educational journey [5]. The demanding curriculum, heavy workloads, and long hours of study create a highly competitive and pressurized environment [3]. Additionally, the emotional strain of dealing with illness, suffering, and death can be emotionally taxing [5]. The needs to maintain the high level of academic performance, pass rigorous exams, and succeed in clinical rotations further contribute to the stress burden experienced by medical students [4]. The relentless pressure and stressors faced by medical students can result in a range of negative effects on their mental well-being [6]. Studies have indicated higher rates of anxiety, depression, burnout, and suicidal ideation among medical students compared to their peers in other disciplines [6]. The constant juggling of academics, clinical responsibilities, and personal life can lead to feelings of overwhelm, isolation, and inadequacy, exacerbating mental health issues [5]. In response to the tremendous stress and pressure, some medical students may turn to maladaptive coping mechanisms, including substance abuse [7]. The accessibility of prescription medications and the culture surrounding stress and self-medication in the medical field pose additional challenges [7]. Substance abuse, whether through prescription drugs, alcohol, or recreational drugs, not only aggravates mental health issues but can also have severe consequences for personal and professional development [6]. Research from India found that the incidence of drug usage among medical students, interns, and house doctors ranged from 32.5% to 81.2% [6]. According to the literature, the transition from school to college predisposes adolescents to engage in drug-using behaviors such as substance start and maintenance [7]. Longitudinal research indicated that smoking, regular alcohol use, a history of alcohol-related issues, anxiety, or rage, and frequent use of alcohol in non-social situations among medical students throughout the undergraduate (UG) course serve as risk factors for future alcohol dependence [8]. The mental health status and substance abuse risk among medical students are pressing concerns that necessitate attention and action [8]. Understanding the specific stressors and pressures inherent in medical education allows for targeted interventions to support students' mental well-being [9]. Therefore, it is of interest to explore the specific stressors faced by medical students, their potential impact on mental health, and the associated risk of substance abuse.

## Methodology:

A facility-based cross-sectional study was conducted in a tertiary care hospital in Karaikal, South India among all under graduate and post graduate medical students who gave consent for the study. A facility-based cross-sectional study was conducted among under graduate & post graduate medical college students in a tertiary care institute. Students who were willing to participate, from, the first year to final year MBBS students & interns were included in the study. Totally 421 medical students were included in the study. Substance abuse was assessed using pre-tested semi-structured questionnaire. Mental health status among medical students was assessed using General Health Questionnaire-12. Questionnaires were sent through mail & WhatsApp as google form. Data regarding substance abuse & mental health status were collected through Microsoft excel sheet and analyzed using SPSS software. Substance abuse was assessed using pre-tested semi-structured questionnaire. Mental health status among medical students was assessed using General Health Questionnaire-12. Categorical variables were represented as percentages & compared using chi-square test. Continuous variables were represented as mean ± 2SD. Univariate & multivariate analysis were done to assess the association between substance abuse, mental health and its risk factors.

## Ethical consideration:

Institute Research Review Board and Vinayaka Mission's Research Foundation Ethical Committee approval was obtained before the commencement of the study. Approval number was VMMC/CM/2023/80.

## Results:

## Socio-demographic profile of study participants:

The majority, 201 (47.7%) participants fell within the age range of 21-23 years, followed by 119 (28.3%) participants in the 17-20 age groups. Gender distribution showed 259 (61.5%) female and 162 (38.5%) male participants. Nuclear families were predominant 334 (79.3%), followed by joint families 62 (14.7%) and three-generation families 25 (5.9%). Undergraduate students constituted the largest category 395 (93.8%) while postgraduate students and faculty comprised 16 (3.8%) and 10 (2.4%) respectively. Among undergraduates, participants were distributed across academic levels, with internship being the highest 109 (27.6%). Hostellers represented the largest group 241 (57.3%), followed by rental outside with friends 97 (23%), day scholars 20 (4.8%), rental outside with family 18 (4.3%), and those staying alone 45 (10.7%). (Table 1)

## Mental Health status and substance abuse pattern:

The majority of participants, 331 (78.6%) did not report substance abuse. About 90 (21.4%) admitted to substance abuse. Similarly, 87 (20.7%) of the participants experienced psychological distress, while 334 (79.3%) reported no such distress. Furthermore, an analysis of the relationship between substance abuse and psychological distress showed a statistically significant association (p = 0.005) (Table 2).

## Factors associated with psychological distress:

The subsequent analysis explored factors associated with psychological distress through unadjusted and adjusted odds ratios. In univariate analysis, gender was not significantly associated with psychological distress. Strikingly, participants aged ≥26 exhibited a significantly lower likelihood of psychological distress (Adjusted OR: 0.3, p=0.001), suggesting a potential protective effect in this age category. While the unadjusted analysis implied at a higher odds ratio for psychological distress in nuclear families, the adjusted results did not reach statistical significance. Substance abuse emerged as a significant predictor of psychological distress in the unadjusted model (OR: 2.4), but this association lost significance after adjustment (p=0.6). No significant associations were observed between psychological distress and specific substances (alcohol, smoking, cannabis) after adjusting for other factors. Staying alone and staying with friends exhibited higher unadjusted odds ratios, but these associations did not remain significant after adjustment. A striking association was found between sleep deprivation and psychological distress (OR: 24.8, p=0.0001), underscoring the importance of sleep-in mental well-being. (Table 3)

## Factors associated with substance abuse:

A similar detailed analysis was conducted for factors associated with substance abuse. Males displayed a significantly higher likelihood of substance abuse compared to females (Adjusted OR: 2.3, p = 0.001), indicating a gender-based disparity. In univariate analysis, older age groups (>26) exhibited a progressively higher likelihood of substance abuse (p = 0.01 for 21-23, p = 0.0001 for 24-26, p = 0.006 for >26), suggesting an age-related vulnerability. In adjusted analysis increase in age serve as independent predictor for substance abuse. Postgraduate students and faculty did not show significant associations with substance abuse in the adjusted model. Staying with friends emerged as a significant factor associated with substance abuse (Adjusted OR: 1.8, p = 0.04), while staying with family showed a non-significant negative trend. Similar to psychological distress, sleep deprivation was significantly associated with substance abuse (OR: 2.0, p = 0.01), highlighting the pervasive influence of sleep on substance use patterns. (Table 4)

## Discussion:

The findings of this study revealed the complex interplay between mental health status and substance abuse patterns among medical students in tertiary care. The prevalence of substance abuse and mental health problems among the participants is a cause for concern and warrants further attention from healthcare providers and educational institutions [9]. The significant association between psychological distress and substance misuse highlights the need for comprehensive interventions that address both mental health and substance abuse issues among medical students. The high prevalence of substance abuse (21.4%) and mental health problems (20.7%) is observed [10]. Prior investigations have revealed that the incidence of depression varies, showing rates between 12.7% and 21.5%, or spanning from 20% to 50% [11]. The frequency of psychological distress observed in our findings (20.7%) is below the levels of depression documented in earlier studies from Spain (30.0%) [12] and Saudi Arabia (31%) [13]. Moreover, a meta-analysis found a depression prevalence of 27.2% among medical students [14]. Additionally, the rate of psychological morbidity identified in our analysis was less than those found in the studies conducted by Guthrie *et al.* [15]. Aktekin *et al.* discovered a 48% occurrence of psychological distress among second-year medical students in Turkey [16], while a study among Nepalese medical students reported a lower prevalence of psychological morbidity (21%) [17]. First of all, college students are at a prime age for the onset of many symptoms of mental illnesses. Age emerged as a potential protective factor against psychological distress, with participants aged 26 years and older being less likely to experience mental health problems. Older age groups (24-26 and >26) seem to be associated with a lower likelihood of psychological distress compared to the reference group (17-20), and the association is significant for the >26 age group. This finding suggests that older medical students may have developed coping mechanisms or support systems that mitigate the impact of stress and psychological distress. Understanding the factors that contribute to this age-related resilience could inform the development of interventions tailored to different age groups within the student population [18]. The association between sleep deprivation and both psychological distress and substance abuse is particularly noteworthy. The high odds ratio for sleep deprivation in relation to psychological distress (OR: 24.8) underscores the critical role of adequate sleep in maintaining mental well-being. Similarly, the association between sleep deprivation and substance abuse (OR: 2.0) highlights the need to address sleep patterns as part of comprehensive interventions targeting substance misuse among medical students. Gender differences were also evident in the study, with men being significantly more likely to abuse substances compared to women. This gender disparity in substance abuse patterns calls for gender-specific interventions that take into account the unique risk factors and needs of male medical students. The occurrence of psychological disturbances was found to be higher in females compared to males, aligning with past research that suggests women typically exhibit greater degrees of psychological distress than men within the broader population [19]. A recent study highlighted that female student, experiencing lower levels of social support, may encounter a reduced sense of coherence. This reduction is significantly associated with the presence of psychological distress among medical students, especially within the female demographic [20]. While family structure and living arrangements showed trends in association with psychological distress and substance abuse, the adjusted analysis did not yield statistically significant results. Nevertheless, these findings warrant further exploration to understand the potential influence of family dynamics and social support systems on the mental health and substance use behaviours of medical students. There are certain limitations in the study. The findings of the present study lack external validity and cannot be generalized in other settings because it is a single-centric facility-based study. Temporality could not be established as it is only a cross-sectional assessment. Social desirability bias could occur for self-reported findings like substance abuse.

## Conclusion:

The implications of these findings are significant for the design and implementation of targeted interventions aimed at promoting the mental well-being of medical students. Interventions that address sleep hygiene, stress management, and substance abuse prevention should be prioritized within medical education and healthcare settings. Additionally, future research should focus on longitudinal studies to track changes in mental health and substance abuse patterns over time, as well as the evaluation of intervention programs tailored to the specific needs of medical students.

## Financial support and sponsorship:

NIL

## Figures and Tables

**Table 1:** Socio-demographic details about study participants (N=421)

	**Frequency (n)**	**Percentage (%)**
Age (years)		
17-20	119	28.3
21-23	201	47.7
24-26	73	17.3
≥26	28	6.7
Gender		
Female	259	61.5
Male	162	38.5
Type of family		
Joint	62	14.7
Nuclear	334	79.3
3 generation	25	5.9
Category of participants		
Undergraduate	395	93.8
Post graduate	16	3.8
Faculty	10	2.4
Year of study		
1st MBBS	85	21.5
2ND MBBS	36	9.1
Final part-1	106	26.8
Final part-2	59	14.9
Internship	109	27.6
Place of stay		
Day scholar	20	4.8
Hosteller	241	57.3
Rental outside(friends)	97	23
Rental outside(family)	18	4.3
Stay alone	45	10.7

**Table 2:** Mental Health status and substance abuse pattern among medical students in a tertiary care (N=421)

	**Frequency (n)**		**Percentage (%)**
Substance abuse			
Yes	90		21.4
No	331		78.6
Psychological distress			
Yes	87		20.7
No	334		79.3
	Substance abuse		p value
Psychological distress	No	Yes	0.005*
No	271	63	
Yes	59	28	
*p-value < 0.05 - significant

**Table 3:** Factors associated with psychological distress among medical under graduate students in a tertiary care centre (N=421)

	**Unadjusted OR**	**Adjusted OR**	**p value**
**Gender**			
Male	1.03 (0.6-1.6)	NA	NA
Female	1	NA	NA
Age group (years)			
17-20	1	NA	NA
21-23	0.6 (0.2-1.5)	NA	NA
24-26	0.5 (0.2-1.2)	NA	NA
>26	0.3 (0.1-0.9)	NA	NA
Type of family			
Nuclear	2.2(0.6-7.6)	NA	NA
Joint family	1.3 (0.3-5.2)	NA	NA
3 generation family	1		
Substance abuse			
No	1	1	
Yes	2.4(1.2-3.4)	0.6(0.1-4.0)	0.6
Alcohol use			
No	1	1	
Yes	2.0(1.2-3.4)	1.9(0.4-8.9)	0.3
Smoking			
No	1	1	
Yes	2.6(1.4-4.8)	1.5(0.4-5.4)	0.5
Cannabis			
No	1	1	
Yes	3.7(1.4-9.5)	1.4(0.3-5.7)	0.6
Category of study participants			
Undergraduate	2.3 (0.2-18.5)	NA	NA
Post graduate	4.0 (0.4-41.6)	NA	NA
Faculty	1	NA	NA
Place of stay			
Staying alone	1.015 (0.3-2.8)	NA	NA
Stay with friends	1.381 (0.4-4.0)	NA	NA
Stay with family	1	NA	NA
Sleep deprivation			
Yes	26 (14-47)	24.8 (13.4-45.9)	0.001*
No	1	1	
*p-value < 0.05 - significant; 1 - Reference

**Table 4:** Factors associated with substance abuse among medical under graduate students in a tertiary care centre (N=421)

	**Unadjusted OR**	**Adjusted OR**	**p value**
**Gender**			
Male	2.1 (1.3-3.3)	2.3(1.4-3.9)	
Female	1	1	0.001*
Age group (years)			
17-20	1	1	
21-23	3.0 (1.4-6.5)	2.7(1.2-6.1)	0.01*
24-26	8.5 (3.7-19.4)	8.0(3.2-20.0)	0.001*
>26	9.1 (3.3-25.1)	10.1(1.9-52.4)	0.006*
Type of family			
Nuclear	1	1	
Joint family	1.4 (0.7-2.7)	1.8(0.9-3.7)	0.08
3 generation family	1.1(0.4-3.0)	1.1(0.4-3.2)	0.8
Category of study participants			
Undergraduate	1	1	
Post graduate	2.3 (0.8- 6.5)	1.0(0.1-6.0)	0.9
Faculty	2.5 (0.7-9.3)	1.6(0.1-13.8)	0.6
Place of stay			
Staying alone	1	1	
Stay with friends	2.9 (1.7-5.0)	1.8(1.1-3.2)	0.04*
Stay with family	1.5(0.5-3.9)	0.4(0.1-1.7)	0.2
Sleep deprivation			
Yes	1.8 (1.1-3.2)	2.0 (1.1-3.7)	0.01*
No	1	1	
*p-value < 0.05 - significant; 1 - Reference
